# A preliminary psychometric evaluation of the activity ordering task with a metacognitive facet (AOT-M)

**DOI:** 10.1590/2317-1782/e20240224en

**Published:** 2025-02-28

**Authors:** Nidhi Lalu Jacob, Aysha Rooha, Anjaly S. Nair, Gagan Bajaj, Vinitha Mary George, Jayashree S. Bhat

**Affiliations:** 1 Department of Audiology and Speech Language Pathology, Kasturba Medical College Mangalore, Manipal Academy of Higher Education - Karnataka, Manipal, India.; 2 Division of Biostatistics, Malankara Orthodox Syrian Church Medical College & Hospital Kolenchery - Ernakulam, Kerala, India.; 3 Department of Audiology and Speech Language Pathology, National Institute of Speech & Hearing - Trivandrum, Kerala, India.; 4 Department of Audiology and Speech Language Pathology, Nitte Institute of Speech and Hearing, Nitte deemed to be University Deralakatte - Mangalore, Karnataka, India.

**Keywords:** Aging, Metacognition, Working Memory, Reliability, Validity

## Abstract

**Purpose:**

The Activity Ordering Task with a metacognitive facet (AOT-M) was developed, in our previous work, to address the disconnect between traditional working memory (WM) tasks and everyday WM demands, the lack of culturally sensitive, context-based WM tasks in India and enhance participant engagement. The present study aims to provide preliminary evidence of the AOT-M's psychometric properties among a non-clinical adult population, evaluate its sensitivity to cognitive and metacognitive changes with aging, establish construct validity, ecological validity, concurrent validity and test-retest reliability.

**Methods:**

Ninety neurotypical adults, evenly distributed across three age groups, participated in the study. Descriptive statistics examined the distribution of performance spans and estimation discrepancies across age groups and the age-related statistical differences were evaluated using the Kruskal-Wallis Test. Construct validity was assessed using Rasch analysis, while ecological validity was evaluated with the Multidimensional Assessment of Research in Context (MARC) tool. Concurrent validity with sentence ordering and digit letter ordering tasks, was determined through Pearson’s correlation coefficient and test-retest reliability was assessed using the Intraclass Correlation Coefficient and Bland-Altman plots.

**Results:**

The patterns observed in WM performance spans and estimation discrepancies highlighted the task's sensitivity to aging related cognitive and metacognitive changes. Evidence from the MARC tool substantiated ecological validity, and concurrent validity was demonstrated through significant correlations with established WM tasks. While Rasch analysis supported construct validity, moderate person reliability indicated some limitations in task sensitivity. The AOT-M demonstrated good test-retest reliability.

**Conclusion:**

Overall, the study provides preliminary evidence of the AOT-M’s good psychometric properties within a neurotypical adult sample, suggesting it to be a promising addition to the cognitive communicative toolbox for Speech Language Pathologists.

## INTRODUCTION

### Working memory and metacognition in everyday life

In everyday life, working memory (WM) is essential for real-time information processing, language comprehension, the retention and manipulation of information necessary for executing complex tasks such as learning, reasoning, decision-making and effective social interactions^(1)^. It plays a crucial role in facilitating a wide range of cognitive-communicative activities necessary for navigating daily routines across various phases of adulthood. For instance, in young adults (YA), WM is necessary for managing complex tasks such as following lectures, studying for exams, problem-solving during group projects, participating in workplace conversations and acquiring new skills^(2)^. Among middle-aged adults (MAA), WM is required for handling projects, organizing schedules, meeting deadlines and balancing work with personal obligations. WM facilitates older adults (OA) in managing daily activities such as medication, financial planning, meal preparation and social interactions^(3)^. WM deficits are prevalent across various cognitive-communicative disorders, including dementia, Mild cognitive impairment, aphasia and Traumatic brain injury, significantly impairing essential linguistic functions such as figurative language use, context integration in conversations, narrative coherence, pronoun resolution, comprehension and overall language processing^(4)^. Therefore, understanding and assessing WM across adulthood is fundamental for promoting cognitive resilience, building cognitive reserves and ultimately supporting healthy cognitive aging^(5)^.

Addressing the significance of WM across adulthood, numerous assessments have been developed and validated; however, traditional lab-based cognitive tasks such as Reading Span, Listening Span, Operation Span, Rotation Span, Digit Span and others^(6,7)^ often fail to capture the multifaceted WM demands of everyday life, leading to a lab-life gap^(8)^. Consequently, ecologically valid WM assessments like 'Shopping Mall Task' and 'Overnight Trip Task' have been developed, aiming to better predict functional performance in everyday contexts^(9,10)^. However, these ecologically valid tasks often lack culturally and linguistically relevant stimuli, frequently demand high-end technology and substantial time investment, which restricts their widespread adoption and usability^(11)^. Given these considerations, there remains a notable scarcity of ecologically valid WM assessment measures tailored specifically for evaluating WM in Indian adults.

Another crucial aspect of cognitive measures in general, and WM specifically, is their potential to assess the metacognitive processes associated with them^(12)^. Specifically, metacognitive processes linked with WM significantly influence individuals' awareness of their cognitive abilities, impacting their performance in everyday communication scenarios^(13)^. Metacognition, described as 'thinking about thinking,' is essential across adulthood influencing decision-making, problem-solving, learning, error monitoring, strategy selection and social interactions^(14)^. It supports active learning, critical thinking, reflective judgment and efficient cognitive offloading, crucial for understanding one's cognitive performance^(15)^. Assessing metacognition is vital in clinical and research settings to gauge how individuals perceive and manage their cognitive health, providing insights into their self-awareness of cognitive changes, aiding early detection and interventions to preserve cognitive function and quality of life throughout adulthood. Several laboratory-based cognitive tasks, including visuospatial WM assessments, listening span tasks, n-back tasks, reading span tasks, digit ordering tasks and operation span tasks are increasingly integrating metacognitive components^(12,16,17)^. These WM assessments utilize both offline measures, such as questionnaires and self-report tools, to explore global metacognition and online measures, such as prospective, concurrent, and retrospective measures, to provide dynamic metacognitive insights across cognitive tasks^(18)^. Despite these advancements, there is a dearth of ecologically valid WM tasks that embed comprehensive metacognitive components, specifically tailored to the Indian context.

### Activity ordering task with a metacognitive facet (AOT-M)

Recognizing the need to address the disconnect between traditional WM task performance and everyday WM demands, participant engagement issues, the benefits of incorporating a metacognitive component and the challenges posed by existing context-based tasks that lack ease of utility and cultural sensitivity in the Indian context, the Activity Ordering Task with a metacognitive facet (AOT-M) was developed^(19)^. The AOT-M was designed following the Analysis, Design, Develop, Implementation and Evaluation (ADDIE) instructional design model, which provided a structured framework across five well established phases^(20)^. The initial Analysis phase involved a thorough literature review to identify gaps and research questions. During the Design phase, the task was conceptualized using the Nominal Group Technique and underwent content validation. In the Develop phase, the content-validated script was computerized in collaboration with an animation artist and integrated into SuperLab software. Pilot testing during the Implementation phase further refined its usability. Our previous work details these first four phases of the task development comprehensively^(19)^.

The AOT-M is a progressive span-based assessment ranging from Level 2 to Level 10, featuring two trials per level to allow an additional attempt upon failure. Structured around everyday scenarios, participants must order activities chronologically based on instructions from various sources. This task requires participants to apply an overlearned ordering principle, actively maintaining both activities and timelines in primary memory until completion. As levels progress, the increasing cognitive demands may exceed the capacity of primary memory, requiring information to be stored in secondary memory for subsequent controlled retrieval. Participants use relevant cues, such as activity timelines, to recall details amidst distractions, continuously monitoring and updating WM representations to maintain accuracy. Attentional control processes are engaged to direct attention to task-relevant information and periodically refresh WM contents, ensuring the sequence of activities remains accurate and updated^(21)^. The AOT-M thus captures the intricate interplay of WM processes such as active maintenance, controlled retrieval, monitoring, updating and inhibition. Additionally, the AOT-M incorporates a metacognitive facet where participants predict their WM span before and after completing the task, providing insights into their self-awareness of their performance. In view of the fact that WM facilitates language comprehension, expression and overall communication^(4)^, the AOT-M, which evaluates both WM and associated metacognition, could serve as a valuable tool for speech-language pathologists in cognitive-communicative assessments.

### The present study

Establishing psychometric properties such as reliability and validity is fundamental in developing new measures for clinical practice, education and research, as they ensure confidence in the accuracy and interpretation of assessments^(22,23)^. Reliability ensures consistency and reproducibility across successive administrations whereas validity determines how well an instrument measures the intended construct^(24)^. Typically, this validation process begins with non-clinical samples to establish initial utility before advancing to validation in clinical populations^(25)^.

The evaluation phase of developing the AOT-M, following the ADDIE instructional design model, is designed as a comprehensive series of investigations beginning with non-clinical populations and progressing to clinical populations to assess the psychometric properties of the novel task. This study represents the first investigation in the series, focusing on providing preliminary evidence of the AOT-M's psychometric properties among non-clinical adult population. Specifically, the aims were to evaluate the AOT-M's sensitivity to cognitive and metacognitive changes associated with aging, assess construct validity using Rasch analysis, ascertain ecological validity, establish concurrent validity and evaluate its test-retest reliability.

## METHODS

The present study focuses on the evaluation phase, presenting the initial psychometric properties of the AOT-M on a small, non-clinical adult population. Approval was obtained from the Institutional Ethics Committee (IECKMCMLR-08/2021/263, IECKMCMLR-05/2023/269).

### Participants

In present research, 90 neurotypical adults were recruited through convenience sampling, with equal representation across the three age groups: YA (18-40 years; male=7, female=23), MAA (41-65 years; male=8, female=22) and OA (> 65 years; male=13, female=17). Adults with a score of more than 26 on the Mini-Mental State Examination^(26)^ having no history of neurological or psychological disorders were included as participants for this phase. The socioeconomic status of all participants was determined as middle class using the Modified Kuppuswamy scale^(27)^. Participant’s English proficiency was verified by ensuring a minimum proficiency score of 'seven' on the Language Experience and Proficiency Questionnaire^(28)^. All participants signed and provided informed consent. Detailed demographic information for all participant groups is provided in Table 1.

**Table 1:** Participant characteristics

Attribute	YA (n=5)	MAA (n=5)	OA (n=5)
**Mean Age & Standard deviation**	21.7±2.45 years	51.2±7.20 years	69.1±6.62 years
**Gender**	Male: n=7	Male: n=8	Male: n=13
	Female: n=23	Female: n=22	Female: n=17
**Mean score on Language Experience and Proficiency Questionnaire**	7.83±1.12	8.06±0.82	7.87±0.591
**Mean socioeconomic status score on Modified Kuppuswamy scale**	20.8±4.19	21.9±3.71	21.6±3.76

Caption: YA = Young adults; MAA = Middle-aged adults; OA = Older adults

### Measures

#### AOT-M

**Figure 1:**
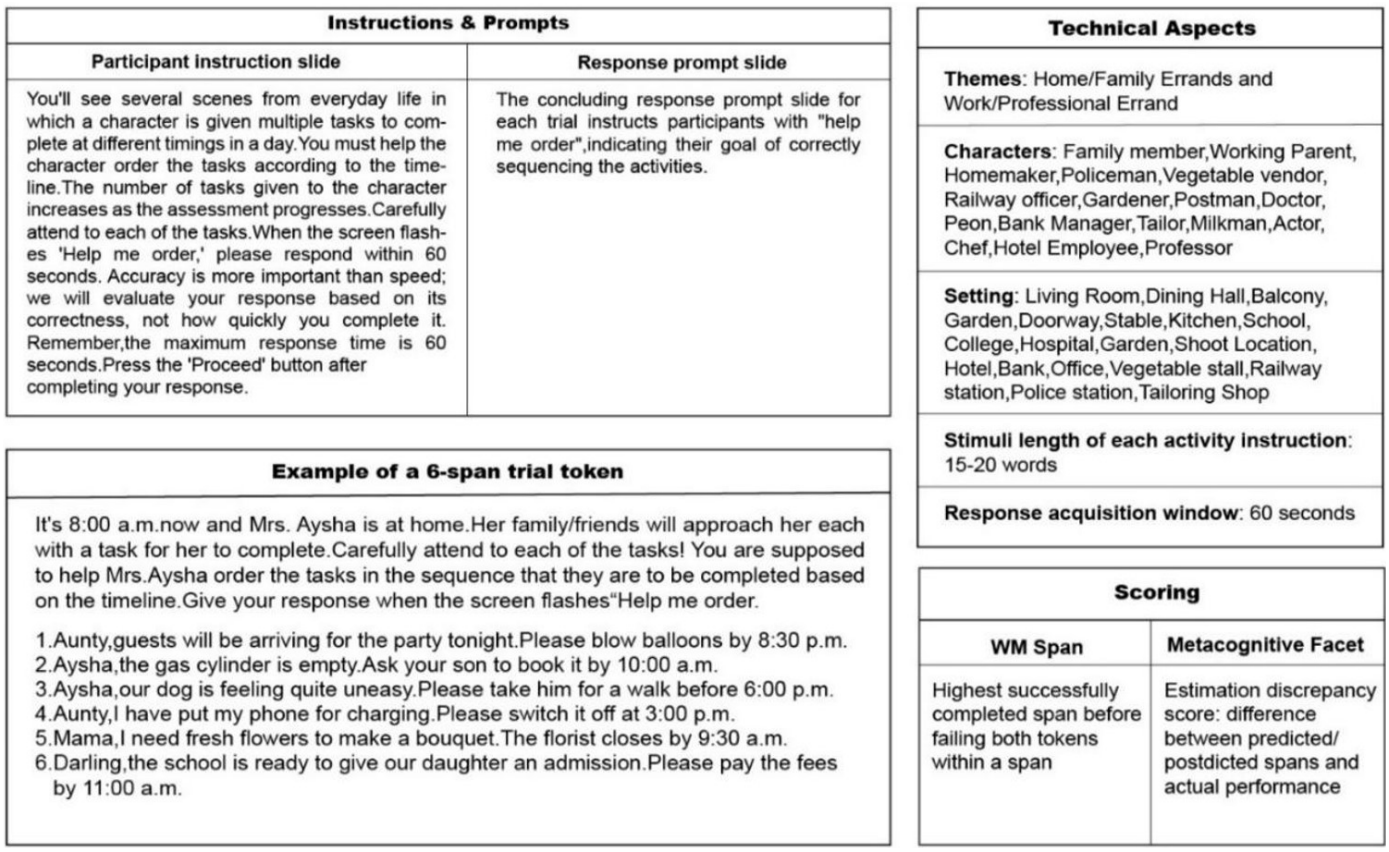
Components of the AOT-M

AOT-M is a span-based WM measure comprising of two components aimed at assessing everyday WM and a metacognitive facet^(19)^. The task, presented in an audiovisual format, involves relatable everyday themes and requires participants to order activities for a character to be completed at various times of the day, based on instructions from family, friends, or colleagues. The primary objective is to arrange these activities in chronological order, from earliest to latest, upon receiving the prompt. The activities range from two to ten, with complexity levels equivalent to the participant's WM span. Each level includes two trials, providing participants a second chance if the initial attempt is unsuccessful. The WM span is recorded as the highest level at which participants can accurately order the activities in chronological order of their timelines. For instance, if a participant successfully orders 4 activities correctly but fails on two trials of 5 activities, their WM span is recorded as 4. An example of a trial is provided in Figure 1, displaying the task components. The metacognitive facet of the AOT-M employs an online method of assessing metacognition, where participants predict their WM span before performing the task and then postdict the highest WM span they believe achieved after completing the task. The metacognitive facet is scored by calculating the estimation discrepancy, which is the difference between the predicted or postdicted spans and the actual task performance span. The AOT-M is administered using SuperLab software, which supports simultaneous auditory-visual presentation with a 1000 millisecond inter-stimulus interval (ISI). The content validation of the AOT-M demonstrated high understandability scores of 90.9% for the script and 89.6% for the task^(19)^.

### Tasks to assess concurrent validity

#### Sentence Ordering (SO) task

SO task is a span-based measure designed to assess WM^(6)^. Participants are required to recall sentences and rearrange the words in increasing order of the word length. This task employs both auditory and visual modalities simultaneously, like AOT-M and presents everyday sentences of varying complexity using SuperLab software. Sentences range from three to ten words, with complexity levels equivalent to the participant's WM span. Each sentence contains words of different lengths in terms of phonemes/letters and syllables, ensuring no two words within a sentence have the same length. Every complexity level, from Level 3 (three-word sentences) to Level 10 (ten-word sentences), is represented by two trials, offering participants a second chance if the initial attempt is unsuccessful with an ISI of 1000 milliseconds. The task is terminated when participants incorrectly perform on two consecutive trials. The WM span is recorded as the highest level at which participants can accurately order words in the target sentence stimuli in ascending order of word length. For instance, if a participant successfully orders a 6-word sentence but fails on two trials of a 7-word sentence, their WM span is recorded as 6. The SO task possesses optimum psychometric properties, including moderate test-retest reliability (Intraclass Correlation Coefficient = 0.559) and strong concurrent validity (r = 0.623, p < 0.001)^(6)^.

#### Digit Letter Ordering (DLO) task

It is an adapted version of the Letter-Number Sequencing task from the Wechsler Adult Intelligence Scale-IV^(29)^, designed to measure WM^(6)^. In this task, participants are presented with a series of letters and digits through both auditory and visual modalities. They are required to first arrange the letters in alphabetical order followed by the digits in ascending numerical order. The task consists of levels ranging from 3 items to 10 items, increasing in complexity. Each stimulus is presented for 2000 ms, with an ISI of 1000 ms. The task is terminated when participants perform incorrectly on two consecutive trials. The WM span is recorded as the highest level up to which the participants can accurately order the items. For instance, if a participant successfully orders a 5-item set of digits and letters but fails on two trials of the 6-item set, their performance span is recorded as 5. The DLO task possesses optimum psychometric properties, including test-retest reliability (Intraclass Correlation Coefficient = 0.619) and strong concurrent validity (r = 0.634, p < 0.001)^(6)^.

### Procedure

**Figure 2:**
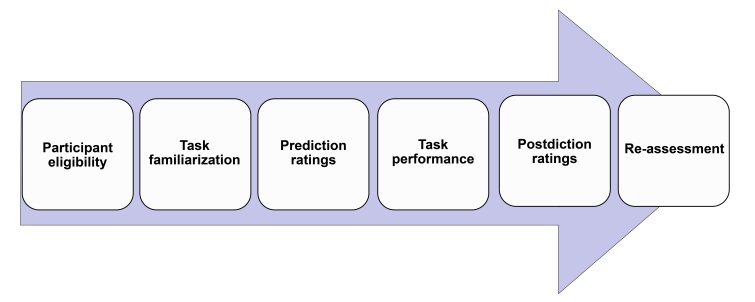
Procedure outline

Initially, information was collected from participants to gather demographic data and ascertain the inclusion criteria. Participants were informed about the study and requested to provide the signed consent form. Participants were then informed that three tasks would be administered: AOT-M, SO and DLO. Task instructions explaining the nature of each task were provided, followed by a maximum of two practice trials to allow for task familiarization. Following task familiarization using practice trials, participants predicted their performance using a Likert scale (1-5), rating their confidence in completing each span. Spans that received ratings of 4 and 5 were considered prediction spans. For example, a rating of 4 for the sixth span in the SO task indicated a prediction span of 6. After making predictions, participants performed the tasks one after the other in random order. WM performance span was assessed based on the highest span correctly ordered by participants in each task. WM performance span was determined by the number of activities correctly ordered on the AOT-M, the number of words correctly ordered on the SO and the number of digits & letters correctly ordered on the DLO. Postdiction ratings were taken using similar questions as the predictions immediately after performing each task. Prediction and postdictions questions are outlined in Table 2. The metacognitive assessment across all three tasks was determined by calculating the estimation discrepancy, which is the difference between the predicted or postdicted spans and the actual task performance span. AOT-M, including its metacognitive facet, was readministered on 10 participants from each age group after a ten-day interval to evaluate test-retest reliability. The sequence of the entire procedure has been outlined in the Figure 2.

**Table 2:** Prediction-postdiction probes

Prediction question	Task	Postdiction question
Up to how many activities do you think you can order correctly?	AOT-M	Up to how many activities do you think you have ordered correctly?
Up to how many word sentences do you think you can order correctly?	SO (George et al., 2020^(6)^)	Up to how many word sentences do you think you have ordered correctly?
Up to how many digits & letters do you think you can order correctly?	DLO (George et al., 2020^(6)^)	Up to how many digits & letters do you think you have ordered correctly?

Caption: AOT-M = Activity ordering task with metacognitive facet; SO = Sentence ordering task; DLO = Digit Letter Ordering task

### Statistical analysis

Statistical analysis was done using SPSS version 26. Descriptive statistics were utilized to examine the distribution of performance spans and estimation discrepancies across various age groups. The Independent-Samples Kruskal-Wallis Test was employed to compare performance spans and estimation discrepancies between the groups. The Chi-square test was used to evaluate differences in gender distribution across the three age groups, while the Mann-Whitney U test was employed to explore the effect of gender on performance spans and estimation discrepancies. The Wilcoxon Signed Ranks Test was used to assess differences between prediction and postdiction estimation discrepancy values. The Kolmogorov-Smirnov test was employed to assess the normality of the data.

### Reliability

Test-retest reliability was assessed using the Intraclass Correlation Coefficient^(23)^. Bland Altman plots were constructed to assess the systematic error (mean difference) and the 95% limits of agreement between the AOT-M initial and subsequent assessment results.

### Validity

#### Ecological validity

The ecological validity was assessed using the Multidimensional Assessment of Research in Context (MARC) tool^(30)^, which evaluates the extent to which psychological and neuroscientific studies capture real-world behavior. MARC tool enables researchers to explicitly report the level of ecological validity by answering seven questions about the study's design, tasks, stimuli, measures, participant sampling and stakeholder involvement. The tool provides a compass plot that visually represents the balance among controlled, partially naturalistic and naturalistic approaches.

#### Construct validity

The construct validity of the WM component of AOT-M was assessed through Rasch analysis to determine whether the task measures the intended construct of everyday WM. Additionally, the analysis aimed to ascertain if the difficulty of the AOT-M increases as task levels progress and whether these levels effectively differentiate individuals with varying WM capacities. A Wright map generated through Rasch analysis allows researchers to visually compare the predicted order of item difficulty with the actual order observed in the dataset.

#### Concurrent validity

The concurrent validity of the AOT-M to measure one’s WM span was assessed by the calculation of the Pearson’s correlation coefficient between the AOT-M and the SO task as well as the DLO task as reference measures. Concurrent validity of the metacognitive facet of AOT-M was assessed by the Pearson’s correlation coefficient between the estimation discrepancies on AOT-M and the SO task as well as the DLO task as reference measures. Concurrent validity was assessed using Pearson’s correlation coefficient^(31)^. Bland Altman plots were constructed to assess the systematic error (mean difference) and the 95% limits of agreement between the performance spans on AOT-M and DLO & SO.

## RESULTS

### Age related cognitive changes on AOT-M

**Figure 3:**
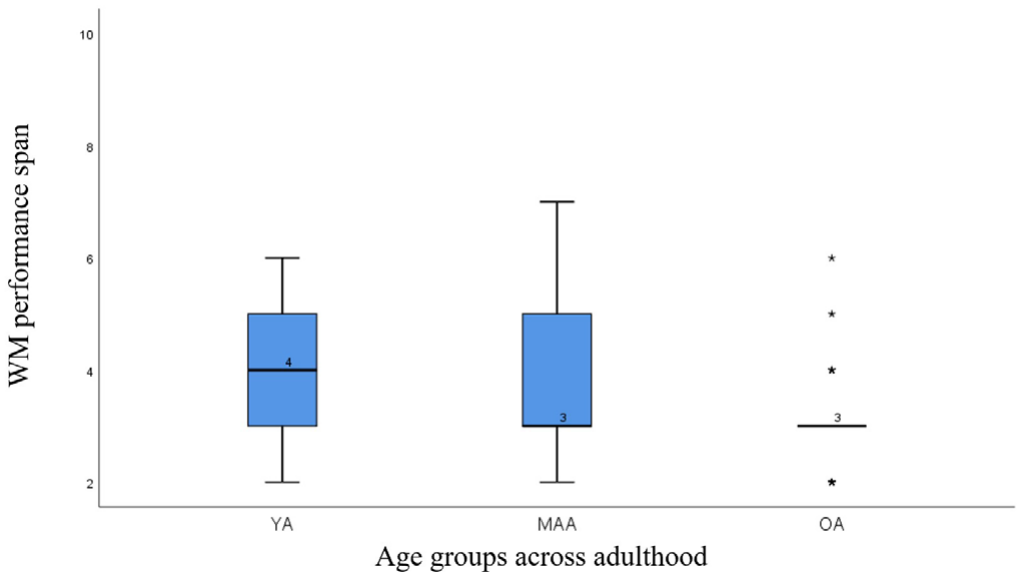
Median performance span on AOT-M across age groups

YA demonstrated the highest performance spans, with a median of 4.00 and interquartile ranges (IQR) spanning from 3.00 (Q1) to 5.00 (Q3) as compared to MAA who showed a slight decline, with a median of 3.00 and IQR values ranging from 3.00 (Q1) to 5.00 (Q3). OA exhibited the lowest performance spans, with both the median and Q3 values at 3.00 and Q1 at 2.75. The Kruskal-Wallis test revealed significant differences in performance spans across the three age groups (H = 11.002, p = 0.004). Subsequent Dunn’s pairwise comparisons highlighted significant differences between MAA and OA (H = 14.217, p = 0.023) and between YA and OAA (H = 20.183, p = 0.001), while the difference between YA and MAA was not significant (H = 5.967, p = 0.34). These findings demonstrate that the AOT-M seems to be sensitive to age-related declines in WM, effectively tapping into cognitive changes associated with aging. The median performance span on AOT-M across various age groups are displayed in the Figure 3. Median and IQR of performance spans on AOT-M are given in Table 3. Chi-square analysis revealed no statistically significant difference in gender distribution across the three age groups (χ^2^ (2, N = 90) = 3.21, p = 0.2). Additionally, no significant gender effect was found on the performance spans (U = 699, p = 0.112). Females exhibited a median performance span of 3.00 (IQR: 3.00 to 5.00), while males reported a median performance span of 3.00 (IQR: 3.00 to 3.75).

Caption: AOT-M = Activity ordering task with metacognitive facet; YA = Young adults; MAA = Middle-aged adults; OA = Older adults; WM= Working memory; * indicates outliers

**Table 3:** Median and interquartile ranges of performance span, prediction and postdiction estimation discrepancy values on AOT-M

Variable	Age Group	Q1	Median	Q3	Kruskal- Wallis H statistic	*p*-value	Comparison group	Kruskal- Wallis H statistic	*p*-value
**AOT-M performance span**	YA	3	4	5	11.002	0.004*	YA vs MAA	5.967	0.34
	MAA	3	3	5			MAA vs OA	14.217	0.023*
	OA	2.75	3	3			YA vs OA	20.183	0.001*
**AOT-M prediction estimation discrepancy**	YA	0	2	3	7.148	0.028*	YA vs MAA	-17.25	0.009*
	MAA	2	3	4.25			MAA vs OA	5.1	0.442
	OA	1.75	2.5	3			YA vs OA	-12.15	0.067
**AOT-M postdiction estimation discrepancy**	YA	-1	0	1	0.851	0.654			
	MAA	-0.25	0	1					
	OA	0	0	1					

* Indicates statistically significant p-value (p < 0.05)

Caption: AOT-M = Activity ordering task with metacognitive facet; YA = Young adults; MAA = Middle-aged adults; OA = Older adults;

### Age related metacognitive changes on AOT-M

The estimation discrepancies on the prediction/postdiction spans (prediction/postdiction span-performance span) on AOT-M were compared between groups:

***Prediction Estimation Discrepancy:*** Significant differences were found in prediction estimation discrepancies across age groups (H = 7.148, p = 0.028). Dunn’s pairwise tests revealed significant differences between YA and MAA (H = -17.250, p = 0.009). YA showed a median discrepancy of 2.00 (IQR: 0.00 to 3.00), MAA exhibited a higher median discrepancy of 3.00 (IQR: 2.00 to 4.25) and OA had a median discrepancy of 2.50 (IQR: 1.75 to 3.00). These results indicate that MAA & OA tend to misestimate their performance more than YA, highlighting age-related differences in prediction estimation accuracy on AOT-M. No significant gender effect was found on prediction estimation discrepancies (U =850, p = 0.873). Females exhibited a median prediction estimation discrepancy of 2.00 (IQR: 1.00 to 3.25), whereas males demonstrated a median prediction estimation discrepancy of 2.00 (IQR: 2.00 to 3.00);
***Postdiction Estimation Discrepancy:*** No significant differences were observed in postdiction estimation discrepancies across age groups (H = 0.851, p = 0.654). YA had a median discrepancy of 0.00 (IQR: -1.00 to 1.00), MAA had a median discrepancy of 0.00 (IQR: -0.25 to 1.00) and OA showed a median discrepancy of 0.00 (IQR: 0.00 to 1.00). Median and IQR of prediction and postdiction estimation discrepancies on AOT-M are given in Table 3. No significant gender effect was found on postdiction estimation discrepancies (U = 699, p = 0.112) (U =850, p = 0.873) (U=857.5, p = 0.922). Both females and males demonstrated a median postdiction estimation discrepancy of 0.00 (IQR: 0.00 to 1.00).

### Reliability

The ***test-retest reliability*** of the AOT-M was evaluated by readministering on 10 participants from each age group after a ten-day interval. The results indicated excellent reliability for AOT-M performance span (Intraclass Correlation Coefficient = 0.966), good reliability for prediction estimation discrepancy (Intraclass Correlation Coefficient = 0.739) and postdiction estimation discrepancy (Intraclass Correlation Coefficient = 0.454). The Bland-Altman plot, illustrating the mean difference (systematic error) and 95% limits of agreement between the initial and subsequent assessment of AOT-M performance spans, is provided as Figure A in the Supplementary Material. Standardized administration and scoring procedures were followed to ensure consistent task execution, including the initiation, termination and scoring of the WM span, as well as the assessment of the metacognitive facet. Therefore, inter-rater reliability was not assessed.

### Ecological validity

**Figure 4:**
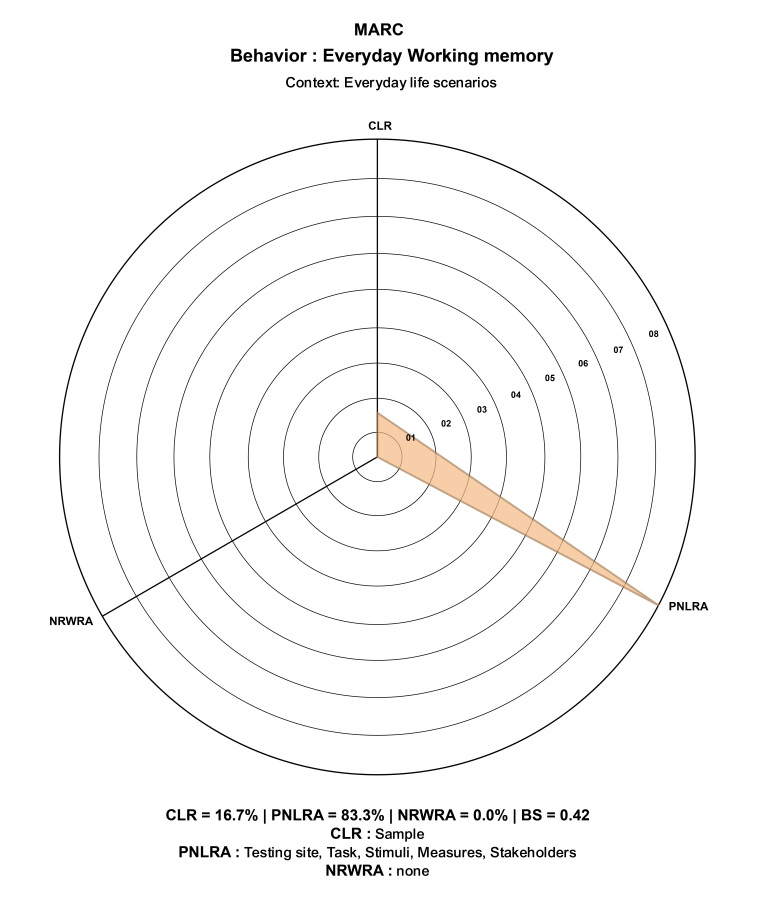
Compass Plot Illustrating Ecological Validity Assessment Using the MARC Tool

The ecological validity was assessed using the MARC tool which generated a compass plot with the following results: 16.7% Controlled Laboratory Research, 83.3% Partially Naturalistic Laboratory Research Approach, and 0.0% Naturalistic Real-World Research Approach. The balance score was calculated to be 0.42, indicating a predominant focus on the partially naturalistic laboratory research approach. The generated compass plot is illustrated in Figure 4.

Caption: CLR = Controlled laboratory research; PNLRA = Partially naturalistic laboratory research approach; NRWRA = Naturalistic real-world research approach; BS = Balance score

### Construct validity

The Rasch analysis was conducted to determine the construct validity of the AOT-M by verifying whether it accurately measures the intended construct of everyday WM and by assessing the increase in difficulty of test items as the levels progress.

**Figure 5:**
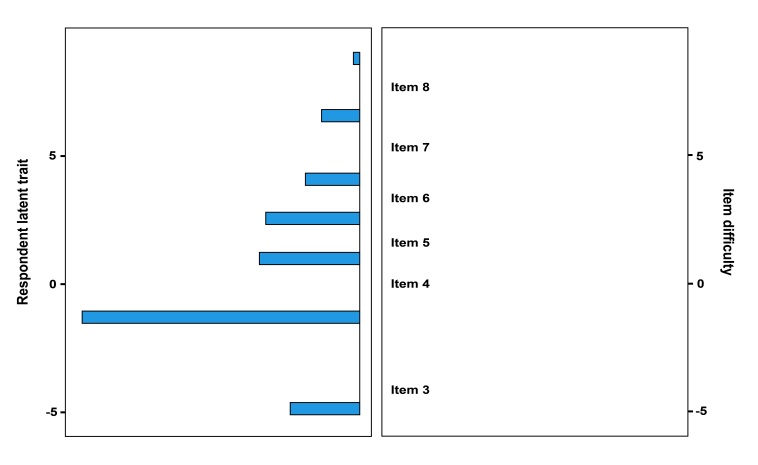
Wright Map illustrating respondent latent trait (WM performance spans) and corresponding item difficulty levels

The mean of the absolute values of the centered Q3 statistic (MADaQ3) was 0.0712, with a corresponding p-value of 0.355, indicating a good fit to the Rasch model and ascertaining that the AOT-M measures the intended construct. The Q3 correlations, which evaluated the independence of the test items within the AOT-M, revealed low correlations among items, indicating that each test item appears to be distinct. All items had infit values within the acceptable range (0.50 to 1.50)^(32),^ indicating that they fit the model well. While the outfit values for items corresponding to spans 3 and 4 were within the acceptable range, items corresponding to spans greater than 4 had notably low values. These low outfit values for higher span items potentially limit the task's ability to differentiate between higher levels of WM capacity. The item statistics revealed a progressive range of measures, from -4.095 to 7.39, indicating that the difficulty of test items increased systematically as levels advanced from span 3 to span 8, as shown in Table 4. This progressive variability in item difficulty can also be appreciated in the Wright map presented in Figure 5. It provides a visual representation of the distribution of respondent latent traits and item difficulties. The left panel of the map shows the distribution of participants' WM performance spans, while the right panel illustrates the item difficulty levels of the various spans. The map indicates that the difficulty level of the spans increases as expected, as fewer participants achieve higher spans. An item reliability of 0.984 was obtained indicating an excellent level of consistency in the item hierarchy however, the person reliability coefficient of 0.606 indicated moderate but inadequate reliability, falling below the accepted threshold of 0.8^(32)^.

**Table 4:** Item Statistics

Test item	WM span	Proportion	Measure	S.E. Measure	Infit	Outfit
Item 3	3	0.8889	-4.095	0.423	0.979	0.613
Item 4	4	0.4111	0.583	0.315	0.811	0.610
Item 5	5	0.2667	1.996	0.348	0.693	0.373
Item 6	6	0.1333	3.731	0.423	0.723	0.273
Item 7	7	0.0556	5.326	0.557	0.766	0.174
Item 8	8	0.0111	7.39	1.057	0.947	0.104

Caption: S.E. = standard error; Infit = Information-weighted mean square statistic; Outfit = Outlier-sensitive means square statistic

Test items 2, 9, and 10 were excluded from the Rasch analysis. Item 2 had uniform responses from all participants, indicating it did not differentiate between different abilities in a dichotomous model. Items 9 and 10 were excluded because no participant could complete them, suggesting they were either too easy or too difficult for the sample, and thus did not contribute meaningful information to the model.

### Concurrent validity

Concurrent validity of the performance span and metacognitive facet of the AOT-M was assessed using Pearson’s correlation coefficients with the SO and DLO tasks. Moderate-strong significant positive correlations were found between the WM span obtained on the AOT-M and SO tasks (Overall: r = 0.410, p < 0.001; YA: r = 0.644, p < 0.001; MAA: r = 0.407, p = 0.019; OA: r = 0.545, p = 0.001). Similarly, moderately significant positive correlations were found between performance spans on AOT-M and DLO scores across all age groups (Overall: r = 0.412, p < 0.001; YA: r = 0.423, p = 0.014; MAA: r = 0.502, p = 0.003; OA: r = 0.695, p < 0.001).

Significant moderate-strong positive correlations were observed between prediction estimation discrepancies on AOT-M and SO across all age groups (Overall: r = 0.615, p < 0.001; YA: r = 0.541, p = 0.002; MAA: r = 0.591, p < 0.001; OA: r = 0.701, p < 0.001). However, correlations between postdiction estimation discrepancies on AOT-M and SO tasks showed mixed results, with significant correlations found in the YA and MAA groups (YA: r = 0.280, p = 0.008; MAA: r = 0.390, p = 0.033), while no significant correlation was observed in the OA group (r = 0.144, p = 0.449). No significant correlations between prediction/postdiction estimation discrepancies on AOT-M and DLO were obtained. The median and IQR of prediction and postdiction estimation discrepancy values on the DLO and SO tasks are provided in Table A in the Supplementary Material. The Bland-Altman plots illustrating the mean difference (systematic error) and 95% limits of agreement between AOT-M and SO (Figure B), as well as AOT-M and DLO (Figure C) are provided in the Supplementary Material.

## DISCUSSION

The present study aimed to provide preliminary evidence on the psychometric properties of the AOT-M. Testing a cognitive task in a neurotypical sample is crucial for assessing feasibility and validity thereby, establishing a necessary foundation before its application to clinical populations^(33)^. Therefore, as an initial step, the AOT-M was administered to a non-clinical sample across adulthood.

The age-related trends on the performance spans on AOT-M suggested that the novel task could examine the age-related differences. The decline in performance spans with age aligns with literature indicating age-related declines in WM^(34)^. As individuals age, increased cognitive effort may be necessary for task engagement, leading to faster depletion of cognitive resources^(35)^. This depletion likely contributes to the decline in performance spans observed on the AOT-M, highlighting its sensitivity to age-related cognitive changes.

The observed pattern of prediction estimation discrepancies across age groups reveals a significant decline in prediction accuracy with age. As individuals transition from young adulthood to middle adulthood, the tendency to misestimate cognitive abilities increases^(36)^. The trends from the present study indicate that the extent of misestimation is higher among MAA and OA compared to YA. These findings align with existing literature that highlights age-related declines in metacognitive accuracy, metacognitive sensitivity and efficiency and increases in metacognitive bias^(14,35)^. The consistent stability of postdiction estimation discrepancies across adulthood aligns with findings indicating preserved metacognitive abilities in aging on retrospective measures^(37)^. Lower estimation discrepancies on postdictions compared to predictions may result from better awareness of performance, enabling more precise calibration. This observation is consistent with literature suggesting that retrospective measures of metacognition tend to be more accurate than prospective measures^(38)^. The decline in prediction accuracy and consistent postdiction estimation discrepancies across adulthood observed in AOT-M highlight its potential to reflect age-related changes in metacognitive abilities.

Studies on gender differences in WM present mixed findings, with some reporting significant effects^(39)^ and others finding no such differences^(40)^. Although the present study did not observe gender effect, drawing meaningful conclusions about gender differences may be constrained by the uneven gender representation of participants, as this was not the primary focus of the research.

Understanding the critical need to assess the stability of cognitive assessments designed for repeated use over time^(22)^, the study evaluated the test-retest reliability of the AOT-M. The results indicate good test-retest reliability indicating consistency and stability over repeated administrations for both the WM component and the metacognitive facet. The Bland-Altman plots demonstrated a reasonable alignment between the WM performance spans across repeated administrations, further reinforcing the test-retest reliability of the AOT-M.

The compass plot and balance score from the MARC tool demonstrate that the AOT-M predominantly adopts a partially naturalistic laboratory approach to simulate everyday WM demands. Stimuli comprising of culturally relevant voice-overs in animated videos depicting scenarios like Home/Family Errands and Work/Professional Errands enhance participant engagement and realism. The task's design, which requires participants to prioritize and order activities based on timelines, mirrors the complex WM demands encountered in daily life. Stakeholder involvement across conceptualization, design and implementation phases further validates the task's relevance and applicability to everyday contexts. Taken together, these elements highlight the AOT-M's capacity to effectively replicate everyday scenarios, substantiating its ecological validity.

Rasch analysis was employed to assess the construct validity of the WM component in AOT-M, providing essential indicators to determine whether the task accurately measures the intended construct and aligns with theoretical predictions. The MADaQ3 value was low, with a non-significant p-value, indicating a good fit between the AOT-M data and the Rasch model^(41)^. The Q3 correlations evaluated the independence of items within the AOT-M. Low Q3 correlations among items indicated that each item measures distinct aspects of WM processes independently. The absence of significant misfit and the presence of low item correlations collectively demonstrate that the AOT-M is psychometrically sound in terms of model fit and item independence.

The difficulty of the test items varied in difficulty, ranging from very easy (e.g., Item 3 with a Measure of -4.095) to very difficult (e.g., Item 8 with a Measure of 7.390). Infit values, which assess how well responses to appropriately challenging items align with the Rasch model, generally fall within the optimal range of 0.5 to 1.50^(32)^, indicating a good fit. Conversely, outfit values consistently below the acceptable range across the test items suggest potential overfitting, where items fit the model better than expected. This overfit appears to be linked to an observed performance plateau, where majority of the participants failed to advance beyond initial task levels. Consequently, responses concentrated at lower difficulty levels reduced variability in participant performance and causing the statistical model to closely fit the data at these easier levels.

The Wright map demonstrated a gradual increase in test item difficulty levels, yet it reveals a notable number of respondents achieving spans of 3 and 4. This suggests that while the items are well-ordered in terms of difficulty, there may be a need to refine the task to better differentiate higher levels of WM capacity. The AOT-M task demonstrates a high item reliability value of 0.984, indicating well-defined and stable item difficulty. However, the person reliability coefficient of 0.606 fell below the accepted threshold of 0.8^(32)^, suggesting inadequate consistency in distinguishing individuals based on their WM capacities. A performance plateau was observed at the initial levels of the task, likely attributable to the demanding nature imposed by the brief ISI of 1000 ms and the recall response format. Despite adaptation from established WM tasks^(6,7)^, this ISI might not have fully met the AOT-M's unique demands, affecting participant performance. Moreover, the recall format of the task could have imposed higher demands than recognition tasks, potentially depleting cognitive resources and impairing WM processing^(4)^. The reduced variability in WM spans among participants might have led to clustering around similar performance levels, thereby creating the impression of more items than participants and consequently contributing to the observed lower person reliability.

The results of the current study support the concurrent validity of the AOT-M, especially its WM component. Performance spans on the AOT-M showed moderate to strong significant positive correlations with both the DLO and SO tasks across all age groups, confirming that the AOT-M effectively measures WM capacity as intended. The Bland-Altman plots indicated a reasonable alignment between the WM span on AOT-M and both the DLO & SO tasks, further reaffirming the concurrent validity of the AOT-M's WM component. These findings align with established literature indicating that tasks assessing similar constructs typically yield homogeneous results and exhibit strong correlations, thereby demonstrating good concurrent validity^(42)^.

Regarding the metacognitive facet of the AOT-M, the prediction estimation discrepancies exhibited moderate to strong correlations with the SO task across all age groups. This indicates that participants' ability to predict their performance on the AOT-M aligned with their predictions on the SO task, reinforcing the concurrent validity of the AOT-M in assessing metacognitive abilities. However, no significant correlations were found between prediction estimation discrepancies on the AOT-M with the DLO task. This lack of correlation could be due to the differences in task nature; while DLO involves remembering and ordering finite digits and letters, SO and AOT-M are more similar in requiring participants to remember strings of linguistic items and order them based on certain criteria. Specifically, the SO task involves remembering words and ordering them by length, analogous to the AOT-M's requirement of remembering activities and ordering them by timelines. Additionally, no significant correlations were observed between postdiction estimation discrepancies on the AOT-M and either the DLO or SO tasks. This may be attributed to the generally low performance spans on the AOT-M, leading to sharper calibrations in postdiction estimates as participants become more familiar with the task, reducing variability and resulting in weaker correlations.

This study represents an initial exploration of the psychometric properties of the AOT-M, a novel context-based task designed to assess everyday WM with a metacognitive facet. The observed patterns of WM spans and estimation discrepancies across different adult age groups suggest that the task is sensitive to the cognitive and metacognitive changes related to aging. The MARC tool provided evidence for its ecological validity, emphasizing its capacity to replicate everyday WM demands. Rasch analysis supported the construct validity of the AOT-M, confirming its alignment with theoretical expectations and efficacy in assessing WM. However, the moderate person reliability, possibly influenced by the performance plateau attributed to unforeseen cognitive overload from minor factors in the task design, emphasizing the need for future research to optimize these design elements to improve task sensitivity. Concurrent validity was demonstrated through significant correlations with established tasks such as DLO and SO, validating the AOT-M's ability to assess both WM and metacognitive abilities through performance estimation discrepancies. Moreover, the test-retest reliability demonstrated consistent performance across repeated administrations, ensuring its stability for longitudinal use.

### Limitations and future directions

The present study was conducted on a small sample of neurotypical adults across adulthood, limiting the generalizability of findings. Future research should prioritize expanding the sample size to encompass broader age ranges and diverse demographic profiles, ensuring a more representative participant pool that includes balanced representation from various professional and academic backgrounds. Additionally, future studies could aim for adequate gender representation to more effectively identify any potential gender effects on various measures of AOT-M. Employing stratified sampling techniques in future studies could mitigate potential biases introduced by convenience sampling, thereby enhancing the study's external validity. Minor task design factors, such as the brief ISI and response format, were identified as potential contributors to increased cognitive demands, resulting in a performance plateau at initial task levels. Future research should focus on refining these task parameters to optimize cognitive load management and improve task sensitivity. Additionally, future studies could extend the psychometric evaluation of the refined task to establish age-specific normative data for WM spans and associated metacognitive facets across different demographics and clinical populations.

## CONCLUSION

The present study marks the first step in the comprehensive series of investigations aimed at establishing the psychometric properties of the AOT-M, a novel task designed to assess everyday WM with a metacognitive facet. The study's findings reveal discernible patterns in WM spans and estimation discrepancies across various adult age groups, indicating the task's sensitivity to cognitive and metacognitive changes associated with aging. Preliminary evidence from this study supports the task’s ecological and concurrent validity, as well as its test-retest reliability. While Rasch analysis supports its construct validity in measuring WM, the observed moderate person reliability value indicates minor limitations in the task sensitivity. Future research would focus on further refining the AOT-M and establishing its psychometric properties across diverse neurotypical and clinical populations, ensuring a comprehensive and representative assessment of its utility.

## Supplementary Material

Supplementary material accompanies this paper.

Table A. Median and interquartile ranges of prediction and postdiction estimation discrepancy values on Digit Letter Ordering and Sentence Ordering Tasks

Figure A. The Bland-Altman plot illustrating the agreement between the initial and subsequent assessment of AOT-M performance spans

Figure B. The Bland-Altman plot illustrating the agreement between AOT-M performance span and SO performance span

Figure C. The Bland-Altman plots illustrating the agreement between AOT-M performance span and DLO performance span

This material is available as part of the online article from https://www.scielo.br/j/codas
