# Supplementary material for: A preliminary psychometric evaluation of the activity ordering task with a metacognitive facet (AOT-M)
Source: Codas. 2025 Feb 28;37(3):e20240224. doi: 10.1590/2317-1782/e20240224en (PMC11895772; doi:10.1590/2317-1782/e20240224en)

Supplementary Material

Table A. Median and interquartile ranges of prediction and postdiction estimation discrepancy values on Digit Letter Ordering and Sentence Ordering Tasks

Variable	Age Group	Q1	Median	Q3	Kruskal- Wallis H statistic	*p*-value
**SO prediction estimation discrepancy**	YA	-0.25	0.00	1.00	1.567	0.457
	MAA	0.00	0.00	2.25		
	OA	0.00	0.50	2.00		
**SO postdiction estimation discrepancy**	YA	-1.00	-1.00	0.00	3.144	0.208
	MAA	-1.00	-0.50	1.00		
	OA	-1.00	0.00	1.00		
**DLO prediction estimation discrepancy**	YA	-1.00	0.00	0.00	1.709	0.426
	MAA	-1.00	0.50	2.00		
	OA	-1.00	0.00	1.00		
**DLO postdiction estimation discrepancy**	YA	-1.00	0.00	0.00	4.135	0.127
	MAA	-1.25	-1.00	0.00		
	OA	-1.00	0.00	0.00		

Abbreviations: SO- Sentence Ordering Task; DLO- Digit Letter Ordering Task; YA-Young adults; MAA -Middle-aged adults; OA -Older adults

Figure A. The Bland-Altman plot illustrating the agreement between the initial and subsequent assessment of AOT-M performance spans

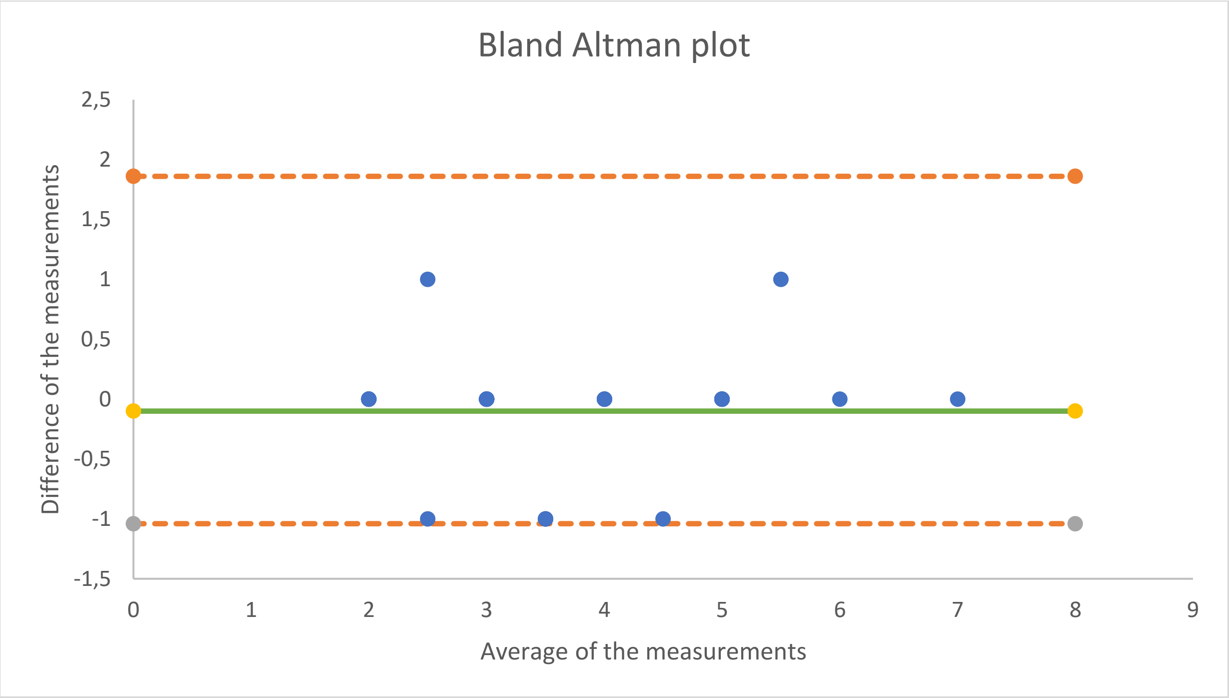

Figure B. The Bland-Altman plot illustrating the agreement between AOT-M performance span and SO performance span

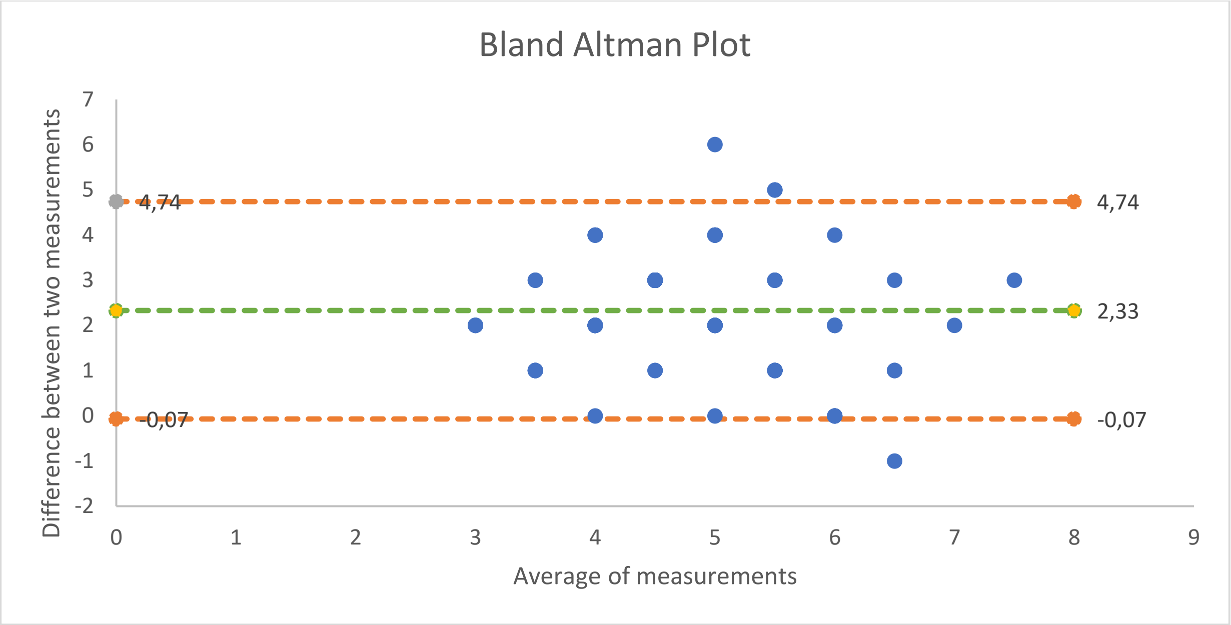

Figure C. The Bland-Altman plots illustrating the agreement between AOT-M performance span and DLO performance span

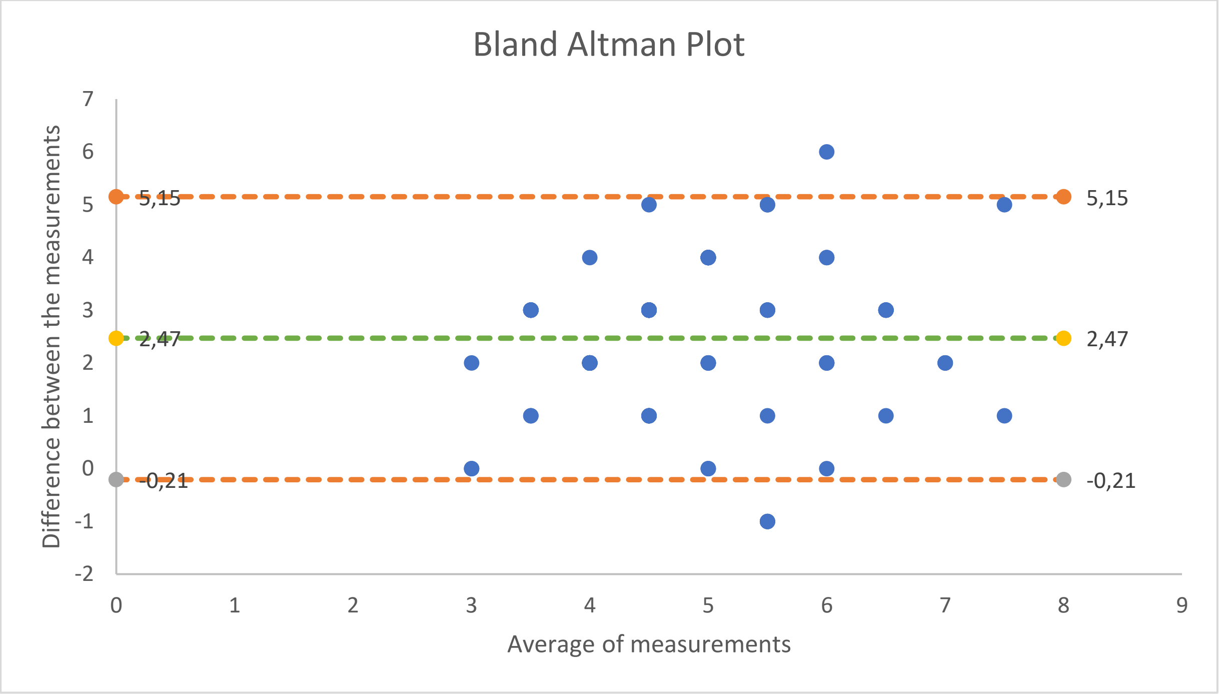

Supplement: Table A. [file codas-37-3-e20240224-suppl01.pdf]
